# Acupuncture for the treatment of marrow suppression after chemotherapy: A protocol for systematic review and meta-analysis

**DOI:** 10.1097/MD.0000000000021876

**Published:** 2020-08-21

**Authors:** Guoyan Geng, Zihan Yin, Mingsheng Sun, Guixing Xu, Jiao Chen, Fanrong Liang, Ling Zhao

**Affiliations:** Chengdu University of Traditional Chinese Medicine, Chengdu, China.

**Keywords:** acupuncture, cancer, chemotherapy, marrow suppression, meta-analysis, protocol, systematic review

## Abstract

**Background:**

Cancer continues to be a severe global health problem and the leading cause of death worldwide. Chemotherapy as the main treatment has various side effects, of which marrow suppression is the most common one. Acupuncture had shown clinical effects for marrow suppression after chemotherapy in many studies. However, the efficacy and safety of acupuncture therapy for marrow suppression after chemotherapy remains unclear.

**Objective:**

This protocol aims to evaluate the efficacy and safety of acupuncture for marrow suppression after chemotherapy according to the existing randomized controlled trials.

**Methods and analysis:**

The randomized controlled trials on acupuncture therapy for marrow suppression after chemotherapy will be searched in the database of Embase, PubMed and Cochrane Library, Allied and Complementary Medicine Database (AMED), Chinese Biomedical Literature Database (CBM), China Science and Technology Journal Database (VIP), China National Knowledge Infrastructure (CNKI), WanFang Database (WF), and related registration platforms (WHO ICTRP, Clinical Trials, and Chinese Clinical Trial Register [ChiCTR]), Grey Literature Database from inception to 1 August 2020. The primary outcomes will be assessed using white blood cell (WBC) count, platelet count, hemoglobin count and the number of neutrophils (N). Review Manager V.5.3 software will be applied for statistical analyses. We will measure the risk of bias of the included studies with Cochrane Collaboration Risk of Bias Tool. Finally, Grades of Recommendation, Assessment, Development, and Evaluation (GRADE) will be used to grade the overall quality of evidence. And we will use the intra-group correlation coefficient to assess the consistency of reviewers.

**Result:**

This systematic review and meta-analysis will put a high-quality synthesis of the efficacy and safety of acupuncture treatment in marrow suppression after chemotherapy.

**Conclusion:**

The conclusion of this systematic review will provide evidence to assess acupuncture therapy is an efficacy and safe intervention to treat and control marrow suppression after chemotherapy.

**PROSPERO registration number:**

PROSPERO CRD42020163336

## 1 Introduction

Cancer is a major public health problem worldwide and is the leading cause of death.^[1,2]^ The lifetime probability of being diagnosed with invasive cancer is approximately 37.7% to 39.3%.^[2]^ Chemotherapy plays an important role in the treatment of cancer in clinical practice.^[3–5]^ As bone marrow is highly sensitive to chemotherapy, marrow suppression (manifested as leukopenia,^[6]^ thrombocytopenia,^[7]^ and anemia,^[8]^ etc) is a major concern in cancer chemotherapy. In addition, marrow suppression could cause fever, skin rash, bone pain, and other adverse reactions.^[9–11]^ Furthermore, it may lead to treatment interruption or even death.^[12,13]^

The main reason for marrow suppression is that chemotherapy cannot only attack tumor cells, but also inhibit the strong proliferation and low differentiation of bone marrow cells and suppress all immature cells with proliferative function, eventually leading to marrow suppression.^[14]^ Currently, treatment options for chemotherapy induced marrow suppression are mainly pharmacological interventions, with high cost and unsatisfactory efficacy, are not ideal.^[15–17]^ Therefore, management of chemotherapy induced marrow suppression is challenging patients, doctors, and health departments of governments. So it is urgent to seek a nonpharmacological intervention for chemotherapy induced marrow suppression. In eastern, acupuncture is an ancient nonpharmacological therapy for chemotherapy induced marrow suppression, with relatively low cost and fewer side effects.^[18]^ Furthermore, increasing clinical studies have shown that acupuncture has a good therapeutic effect for chemotherapy induced marrow suppression.^[19–22]^

Based on these grounds, we have confused an important clinical question: Is acupuncture effective and safe for patients with marrow suppression after chemotherapy? Unfortunately, from the perspective of evidence-based medicine, the efficacy and safety of acupuncture for marrow suppression after chemotherapy is still unclear. Therefore, we have an opportunity to assess the issue and envision this systematic review to explore the efficacy and safety of acupuncture treatment for marrow suppression after chemotherapy.

## 2 Objectives

The purpose of this study is to assess the efficacy and safety of existing acupuncture methods in the treatment of marrow suppression after chemotherapy through systematic review and meta-analysis.

## 3 Methods

This systematic review (SR) will be conducted in accordance with the Cochrane Handbook for Intervention Reviews. Evaluation will be carried out following the items from the Preferred Reporting Items for Systematic Reviews and Meta-Analyses (PRISMA).^[23]^

### 3.1 Patient and public involvement

The public or participants were not covered in the design of this protocol.

### 3.2 Eligibility criteria

#### 3.2.1 Types of studies

All randomized controlled trial (RCTs) about acupuncture for marrow suppression after chemotherapy without language or publication type restriction. Nonrandomized clinical studies, quasi-RCTs, cluster RCTs, and case reports will be excluded.

#### 3.2.2 Types of participants

Trials involving adult patients diagnosed with chemotherapy induced marrow suppression will be included. We will not enroll studies of participants with other specific diseases, and only extract data on adults with chemotherapy induced marrow suppression.

#### 3.2.3 Types of intervention

Acupuncture treatments (manual acupuncture, electroacupuncture, ear acupuncture, warm needling, moxibustion, etc) as monotherapy or additional therapies regardless of stimulation methods and needling techniques will be included in intervention group. Acupuncture therapy combined with blood-letting therapy, cupping, herbal medicine, point injection, or laser acupuncture will be excluded.

#### 3.2.4 Types of control group

Control group will cover conventional medicine, placebo group and sham acupuncture (nonacupoint, minimal), no treatment, usual care, and others. RCTs which compare different technologies/different acupoints will be excluded from our study.

#### 3.2.5 Types of outcome measures

Studies reporting one or more of the following outcomes will be included.

##### 3.2.5.1 Primary outcomes

The main objective of the SR is to evaluate the efficacy and safety of acupuncture for chemotherapy induced marrow suppression; therefore, the primary outcomes are as follows: white blood cell (WBC) counts,^[24]^ platelet counts,^[25]^ hemoglobin counts,^[26]^ or other validated outcome measures.

##### 3.2.5.2 Secondary outcomes

The secondary outcomes include anxiety measured by Self-rating Anxiety Scale,^[27]^ Hamilton Anxiety Scale^[28]^ or others; depression evaluated by Self-rating Depression Scale,^[29]^ Hamilton Depression Scale^[30]^ and the like; quality of life measured by validated scales, like the European Organization for Research and Treatment of Cancer Quality of Life Questionnaire (EORTC QLQ),^[31]^ the Edmonton Symptom Assessment System,^[32]^ Karnofsky Performance Status score;^[33]^ adverse events related to acupuncture (such as nausea, fainting, hematoma, etc).

### 3.3 Search strategy

#### 3.3.1 Electronic searches

From the inception dates to September 1, 2020, the following electronic databases will be searched: PubMed, Embase, Cochrane Library, Allied and Complementary Medicine Database (AMED), Chinese Biomedical Literature Database (CBM), China Science and Technology Journal Database (VIP), China National Knowledge Infrastructure (CNKI), WanFang Database (WF).

#### 3.3.2 Searching other resources

We will search clinical trial registries (WHO ICTRP, Clinical Trials, and Chinese Clinical Trial Register) and Grey Literature Database. Besides, the following journals in China will be searched: *Acupuncture Research*, *Chinese Acupuncture and Moxibustion* and *Journal of Traditional Chinese Medicine*. Additional trials will be further identified according to the list of all identified publications including relevant systematic reviews and meta-analyses.

#### 3.3.3 Searching methods

Although the search methods of different databases are varied, the search terms are mainly composed of 3 parts:

1. clinical condition: cancer, chemotherapy, marrow suppression, etc;
2. acupuncture methods: acupuncture therapy, manual acupuncture, electroacupuncture, ear acupuncture, transcutaneous electrical nerve stimulation and the like;
3. study type is random controlled trial (RCT).

The searching strategy of PubMed is presented in Table 1, and the equivalent words will be used in other sources.

**Table 1:**
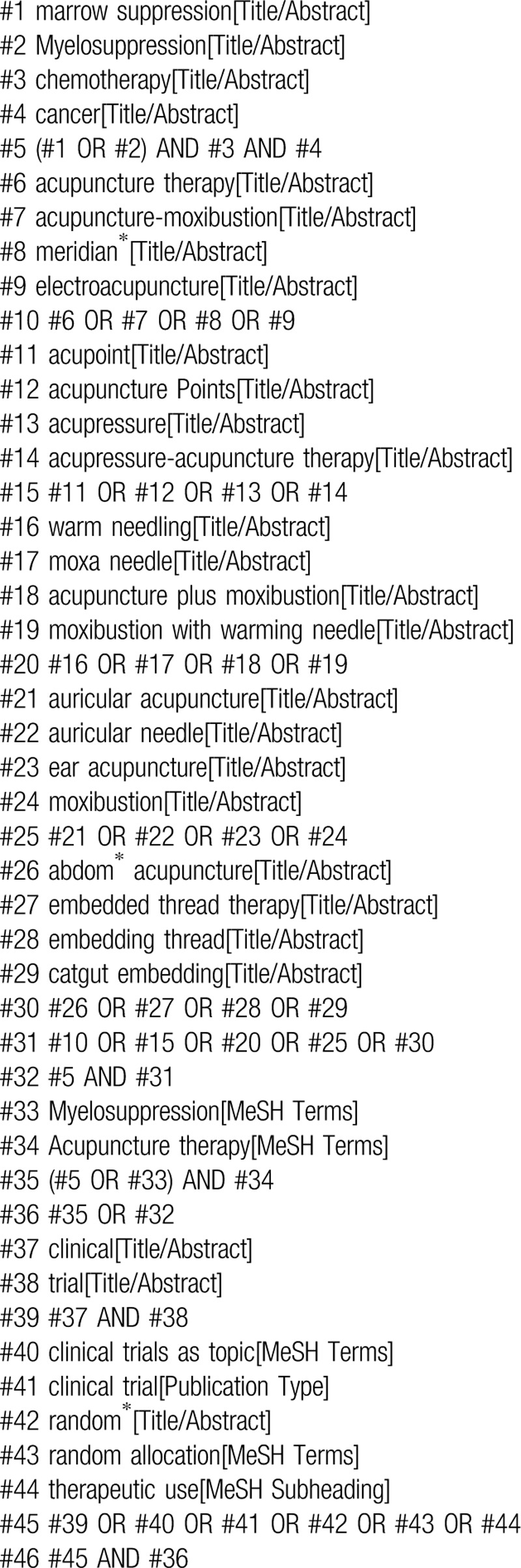
Search strategy for the PubMed database.

### 3.4 Data collection and analysis

#### 3.4.1 Study selection

Two researchers (GG and MS) will have a professional training for this study. And we will conduct the intra-group correlation coefficient to evaluate the consistency between 2 reviewers. When selecting a study, firstly we need to read the title/abstract of the trial to remove duplicate cases and find eligible works. The search results will be uploaded through NoteExpress. GG and MS will independently select through screening the titles and abstracts. Any disagreements in research will be resolved through discussions by 2 reviewers (GG and MS). If they do not come to an agreement, the 3rd party (LZ or FL) need to make the final decision.

#### 3.4.2 Data extraction

**Figure 1:**
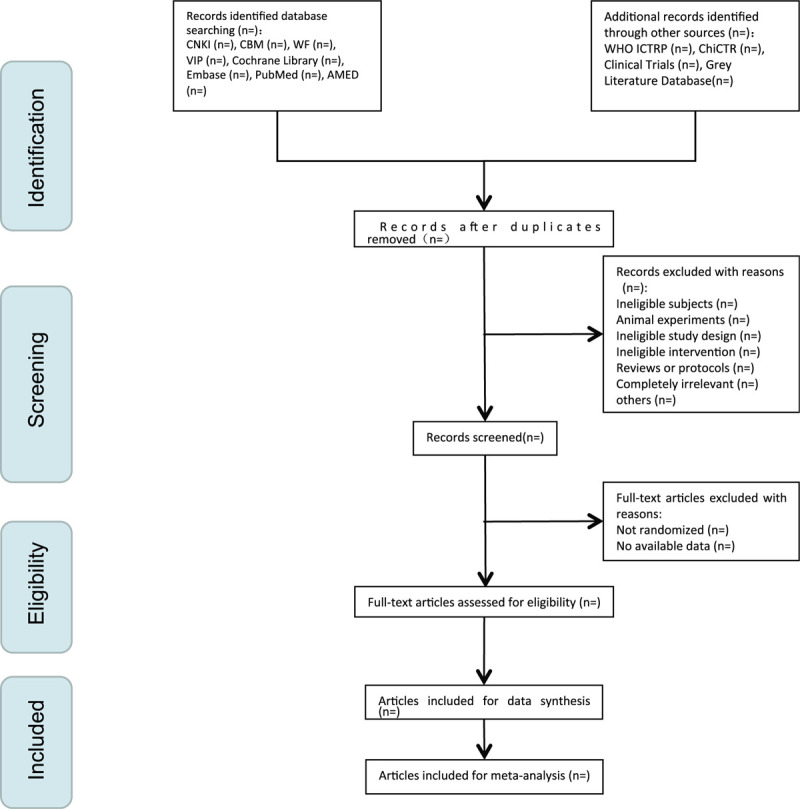
The Preferred Reporting Items for Systematic Reviews and Meta-analyses Protocols (PRISMA-P) flow chart of selection process.

Data extraction will be carried out independently by 2 reviewers (GG and MS) through a standard data extraction form. If there is a disagreement, it will be discussed and judged by the arbitrator (LZ). Two researchers will fill and crosscheck the message into Excel. We will use the intra-group correlation coefficient to assess the consistency of reviewers. General data for selected studies will be extracted, including first author, year of publication, country, age, gender, diagnostic criteria, sample size, treatment group, control group, acupoints, acupuncture details, outcome, conclusion, etc. We will show a Preferred Reporting Items for Systematic Reviews and Meta-analyses Protocols (PRISMA-P) flow chart (Fig. 1) to introduce the selection procedure.

#### 3.4.3 Quality assessment

Two reviewers will independently assess the risk of bias with Cochrane Collaboration's tool,^[34]^ which covers 7 domains: selection bias, performance bias, blinding of participants/personnel, blinding of outcome, attrition bias, reporting bias and other sources biases. It will be classified as “low risk of bias,” “unclear risk of bias” and “high risk of bias.” In case of disagreement, the arbiter (LZ or FL) shall be consulted. Graphic representations about risk of bias will be generated using Review Manager (RevMan) V.5.3.0.

#### 3.4.4 Measures of treatment effect

For continuous data, the mean difference or standard mean difference with 95% confidence intervals will be conducted to estimate it. And the dichotomous information will be calculated by risk ratio with 95% confidence intervals.

#### 3.4.5 Dealing with missing data

When the data of articles are ambiguous or insufficient, we will contact the author to require original information through e-mail or telephone. If it is not available, the impact of missing data will be described and discussed in the study necessarily.

#### 3.4.6 Data synthesis

The data will be conducted and synthesized by the RevMan V.5.3.0 from Cochrane Collaboration. We will choose the fixed effects model (*I*^2^ < 50%) or random effects model (*I*^2^ ≥ 50%). The effect size with risk ratio will be suitable for dichotomous data, and the continuous data will be analyzed by mean difference or standard mean difference. When meta-analysis is not feasible, the results will be described narratively.

#### 3.4.7 Assessment of heterogeneity

We will concentrate on details of patients, interventions, and outcomes to test heterogeneity. According to the Cochrane Handbook V.5.3.0, we will conduct the I^2^ test or the tau^2^ test to assess statistical heterogeneity in each analysis. It is premeditated that it is no heterogeneity between the RCTs when *P* > .1, *I*^2^ < 50%, and the fixed effects model will be used for statistics. Otherwise, when *P*≤.1, *I*^2^≥50%, we adopt random effects model to analyze.

#### 3.4.8 Publication bias

If necessary, meta-regression will be performed to avoid publication bias and symmetry of the funnel plot will be used to assess publication bias across studies or selective reporting bias.

#### 3.4.9 Subgroup analysis and sensitivity analysis

We will perform subgroup analysis based on characteristics of patients, acupuncture methods, outcomes and the like when a substantial heterogeneity is found. Statistical heterogeneity in the study will be quantitatively assessed by *I*^2^. We will make a narrative comment to illustrate this point if the heterogeneity could not be found.

If possible, we will perform a sensitivity analysis to test the robustness of the combined treatment effect. We will consider 2 factors: the effect of high risk of bias or unclear risk of bias, and the effect of the selected model. If inconsistent results are found, use caution when introducing the results and making conclusions.

#### 3.4.10 Grading of quality of evidence

We will eliminate the quality of evidence through Grades of Recommendation, Assessment, Development, and Evaluation (GRADE) approach.^[35,36]^ The GRADE will be carried out to evaluate the quality of evidences from five items limitation (risk of bias, inconsistency, indirectness, imprecision, and report bias) and additional fields where appropriate in it. And the evidence will be ranked as “high’,” “moderate,” “low,” or “very low.”

#### 3.4.11 Patient and public involvement

Nobody will be directly involved. Only data and the sources will be used for this SR.

## 4 Discussion

The NCCN Clinical Practice Guidelines in Oncology (NCCN Guidelines) pointed out that after surgery or inoperable patients, chemotherapy are clearly listed as the first-line treatment strategy for many cancers due to the efficacy.^[37]^ However, chemotherapy cannot only attack tumor cells, but also inhibit the strong proliferation and low differentiation of bone marrow cells and suppress all immature cells with proliferative function, eventually leading to marrow suppression.^[14]^

In China, acupuncture plays an important role in the treatment of chemotherapy induced marrow suppression. However, no SR which is the key part of evidence integration^[38]^ has confirmed that acupuncture is safe and effective in chemotherapy induced marrow suppression and the mechanism of acupuncture in the treatment of chemotherapy induced marrow suppression is complex and unclear. We urgently need to assess the efficacy and safety of acupuncture for chemotherapy induced marrow suppression through SR.

To the best of our knowledge, the study will be the first SR to investigate acupuncture treatment for chemotherapy induced marrow suppression. We expect that the review could provide a basis for acupuncture treatment for chemotherapy induced marrow suppression and offer more and better treatment options for patients. Based on clinical evidence of the SR, it may also provide new treatment options for doctors. However, there may be some limitations (different types of acupuncture, varied control group, lack of research, etc) in the SR, which may lead to potential heterogeneity. Necessarily, sensitivity analysis or subgroup analysis would be adopted to explain the heterogeneity.

## Author contributions

Guoyan Geng and Zihan Yin contributed equally in this paper. Zihan Yin and Ling Zhao conceived this study. Guoyan Geng and Zihan Yin will develop the study protocol and will implement the systematic review under the supervision of Jiao Chen. Guixing Xu will provide the statistical analysis plan of the study and will conduct data analysis. Guoyan Geng and Mingsheng Sun will perform the study search, screening, and extraction of data whereas Fanrong Liang and Ling Zhao will review the work. Guoyan Geng and Zihan Yin wrote the first manuscript draft and all authors gave input to the final draft of the protocol.
